# Improving CT-Derived Fractional Flow Reserve Analysis: A Quality Improvement Initiative

**DOI:** 10.7759/cureus.10835

**Published:** 2020-10-07

**Authors:** Jeffrey Waltz, Madison Kocher, Jacob Kahn, Rebecca Leddy, Jordan H Chamberlin, Daniel Cook, Jeremy R Burt

**Affiliations:** 1 Diagnostic Radiology, Medical University of South Carolina, Charleston, USA; 2 Radiology, Medical University of South Carolina, Charleston, USA; 3 Cardiothoracic Imaging, Medical University of South Carolina, Charleston, USA

**Keywords:** ccta, ffrct, cad-rads, coronary artery disease, quality improvement

## Abstract

Objectives

The aim of this study was to identify factors and quality improvement strategies to improve coronary computed tomography angiography (CCTA) studies referred for fractional flow reserve derived from CT angiography (FFRCT) analysis.

Methods

Thirty randomly selected CCTAs were analyzed for quality control. A uniform CCTA protocol was implemented by an in-house steering committee, emphasizing the importance of adequate heart rate control and nitroglycerine usage. Sixty additional randomly selected CCTAs were evaluated for quality at multiple time points during intervention, and FFRCT acceptance rate was analyzed at the conclusion.

Results

Prior to the implementation of this quality improvement program, our overall institution-specific percent acceptance rate was 76.1% for FFRCT compared to the national average of >95%. Post-intervention, this was improved to an average acceptance rate of 90% for FFRCT analysis.

Conclusions

Establishment and strict adherence to CCTA imaging protocols with appropriate training and adequate buy-in of CT technologists and nurses is a viable way of improving the quality of imaging and subsequent patient care.

## Introduction

Current guidelines recommend the use of non-invasive anatomic imaging, including coronary computed tomography angiography (CCTA), for first-line testing in patients with suspected intermediate-risk stable coronary artery disease (CAD) [1,2]. CCTA and invasive coronary angiography have high accuracy for the detection of coronary artery stenoses based on anatomy evaluation, but they are less helpful for the identification of functionally significant, flow-limiting stenoses of approximately 50-90% [1,3]. Fractional flow reserve derived from CT angiography (FFRCT) has been shown to be an effective adjunct to CCTA in the diagnosis of flow-limiting stenosis and determining prognosis [4]. FFRCT utilization is now being recognized by large insurers who are covering the cost of this added service.

The purpose of our institution’s CCTA-FFRCT program is to improve the accuracy of diagnosis and management of CAD, avoid additional unnecessary procedures, reduce cost, and improve patient and physician satisfaction. Although FFRCT has been shown to be a valuable tool in the evaluation of intermediate-range stenosis on CCTA, the analysis is highly sensitive to scanning protocol and artifacts [4]. It has been shown that image quality of CCTA is closely associated with the heart rate at the time of study acquisition [5]. The rejection rate of FFRCT in the literature ranges from 2% to 33% mainly due to differences in imaging acquisition, study incompletion such as missing best diastolic or systolic reconstructions for myocardial segmentation, patient-specific factors including body habitus and motion, and artifacts including calcium blooming, motion, and low contrast [4,6]. In contradistinction, the rejection rate at our facility of radiologist-referred FFRCT was discovered to be as high as 50%, limiting comprehensive evaluation of coronary artery stenoses and further patient management.

The intention of this project was to evaluate the factors contributing to the high rate of FFRCT rejection at our institution, identify high yield interventions, and assess its effect on the quality of CCTA studies produced as well as the overall acceptance of CCTA studies for FFRCT analysis. Our primary measure was the overall improvement of FFRCT analyses provided in order to maximize yield of the study and improve patient management.

## Materials and methods

Initial assessment

An initial review of a subset of 30 randomly chosen CCTA studies out of a total of 114 studies performed in July and August 2019 was completed by a sub-specialist cardiovascular radiologist to determine the overall quality of the studies in addition to factors limiting the utility of the studies. CCTA studies were assessed according to CAD-RADS criteria, a standardized method of assessing the highest-grade coronary artery lesion for adequacy of study: excellent (no artifacts), good (minor artifact but good diagnostic quality), acceptable (moderate artifacts), or poor/suboptimal (severe artifacts) [7]. The first three categories, excellent, good, and acceptable, were deemed as diagnostic and acceptable for FFRCT evaluation, whereas the last category, poor/suboptimal, was deemed non-diagnostic and therefore unsuitable for FFRCT evaluation referral. A non-diagnostic study was defined as poor quality in one or more coronary artery segments, which would further preclude FFRCT evaluation. In an attempt to optimize the quality of the studies, direct lines of communication were established with the CT technologists and nurses to reveal protocol and acquisition shortcomings. To evaluate potential issues, a question and answer session with a brief didactic lecture was given by an expert cardiovascular radiologist to the CT technologists and the nursing staff, including an overview of coronary artery imaging and the purpose of performing CCTA and FFRCT at our institution. From this discussion with the technologists and nurses, the lead cardiac imager noted several confounding factors described by the staff that were potentially leading to poor image quality. These included a lack of a standardized protocol regarding administration of nitroglycerine and beta-blockers and a lack of understanding of how to assess studies for quality control on the scanner.

The standardized Coronary Artery Disease - Reporting and Data System (CAD-RADS) method of assessment was used to evaluate the final images obtained for all patients. The scoring system ranges from CAD-RADS 0 (complete absence of stenosis) to CAD-RADS 5 (presence of at least one totally occluded coronary artery) and allows for specific recommendations to be included in the radiology impression for appropriate management. All CCTA examinations at our institution with a CAD-RADS of 3 (50-69% stenosis) were referred for FFRCT analysis unless otherwise indicated. Finally, a complete FFRCT analysis was performed on all studies meeting our hospital’s inclusion criteria.

Initial medication trial

To evaluate the effect of pre-scan medication use on overall CCTA image quality at our institution, the radiology nurses were instructed to give every patient nitroglycerine and a beta-blocker, if required and not contraindicated, to achieve a heart rate of <70 and preferably <60. All patients were to receive 0.8 mg of sublingual nitroglycerine and the additional premedication instructions for heart rate (Table 1).

**Table 1:** Metoprolol medication protocol that was taught to the radiology nurses. *Oral medications were to be administered 3-12 hours prior to CCTA. **Metoprolol PRN was to be an IV push of 5 mg metoprolol every 5 minutes up to eight doses for a target heart rate of 60 beats per minute and held for a systolic blood pressure of less than 100 mg. CCTA, coronary computed tomography angiography

Heart rate (beats per minute)	Metoprolol dose and route of administration
>75	Oral metoprolol 100 mg*; IV metoprolol PRN**
66-75	Oral metoprolol 50 mg*; IV metoprolol PRN**
55-65	Oral metoprolol 25 mg*; IV metoprolol PRN**
<54	No medication needed

**Figure 1:**
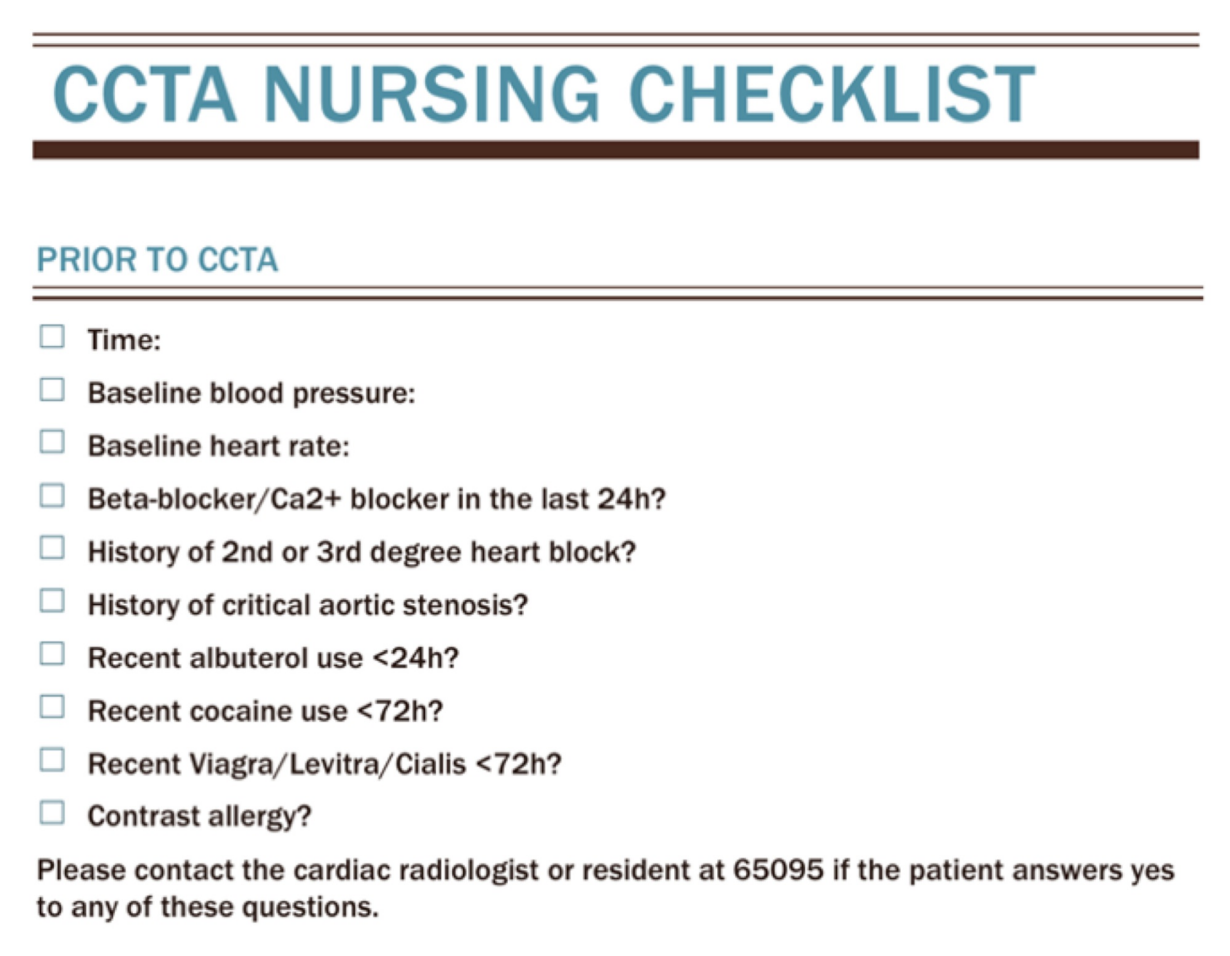
Nursing checklist provided to the radiology nurses to be completed prior to nitroglycerine and metoprolol administration for the CCTA examination. CCTA, coronary computed tomography angiography

The nurses were then instructed to contact the cardiovascular radiologist on call if there was a contraindication to heart rate control or nitroglycerine administration. A list of contraindications for both nitroglycerine and beta-blocker administration was provided (Figure 1).

A second random sample of 30 CCTA cases performed during the month of November 2019 was reviewed by the same expert cardiovascular radiologist to assess for early post-intervention changes following the initial medication changes to confirm that improvement could be seen in the visual quality score.

Standardized protocol creation and implementation

An in-house steering committee was created comprising radiology administration, cardiovascular radiologists, cardiologists, an emergency medicine physician, CT technologists, and radiology nursing staff. The purpose of the committee was to make the CCTA acquisition protocol uniform. A proposed imaging pathway was developed with consensus by the steering committee members, which emphasized the need for consistent medication administration prior to scanning. An electronic order set was created within our electronic medical record to aid in the consistency of this protocol.

The steering committee recommended that one-on-one training be provided to the technologists regarding appropriate scanning parameters, contrast dosages, and injection rate (Table 2). Monthly meetings were conducted by the same cardiovascular radiologist with the head technologists to discuss problem areas and concerns.

**Table 2:** Checklist for appropriate CT imaging acquisition parameters.

Siemens Force Dual Source CT; image during inspiration – acquire imaging from the carina through apex of the heart; run a test bolus; most patients will receive retrospective gating for function and also get coronary artery calcium scan	
kVp	Care kV (ref. kV is 100 used for average-sized patients)
Effective mAs	CareDose (ref mAs 288)
Rotation time	0.25 seconds
Acquisition time	Heart rate dependent
Collimation	192 x 0.6 mm
Pitch value	Heart rate dependent
Scan direction	Craniocaudal
ECG dose modulation	Used for all retrospective scans (Siemens MinDose)
Contrast injection rate	Based on BMI (range 4-8 mL/seconds) (BMI < 35: 4; BMI > 35: 5; BMI > 40: 6; BMI > 45: 7; BMI > 50: 8)
Other parameters: warm contrast helps with high flow rates; patients must have large bore IV for high-flow rates (18G); if IV is in the accessory cephalic vein of the forearm, do not use >20G; contrast dose depends on flow rate, which depends on BMI; always follow contrast injection with saline chaser (50-100 mL); contrast used is Omnipaque® 350 mg/mL.	

A third and final set of 30 random CCTA cases were reviewed during the month of February 2020 for their visual quality score. Power calculations were performed using XLSTAT 2020.1.2 (Addinsoft, New York, NY, USA). Correlation testing and visualization were performed using R v3.6.3 (R Foundation for Statistical Computing, Vienna, Austria; https://www.R-project.org/) and the ggpubr package (ggpubr: 'ggplot2' Based Publication Ready Plots, R package version 0.3.0; https://CRAN.R-project.org/package=ggpubr), respectively.

**Figure 2:**
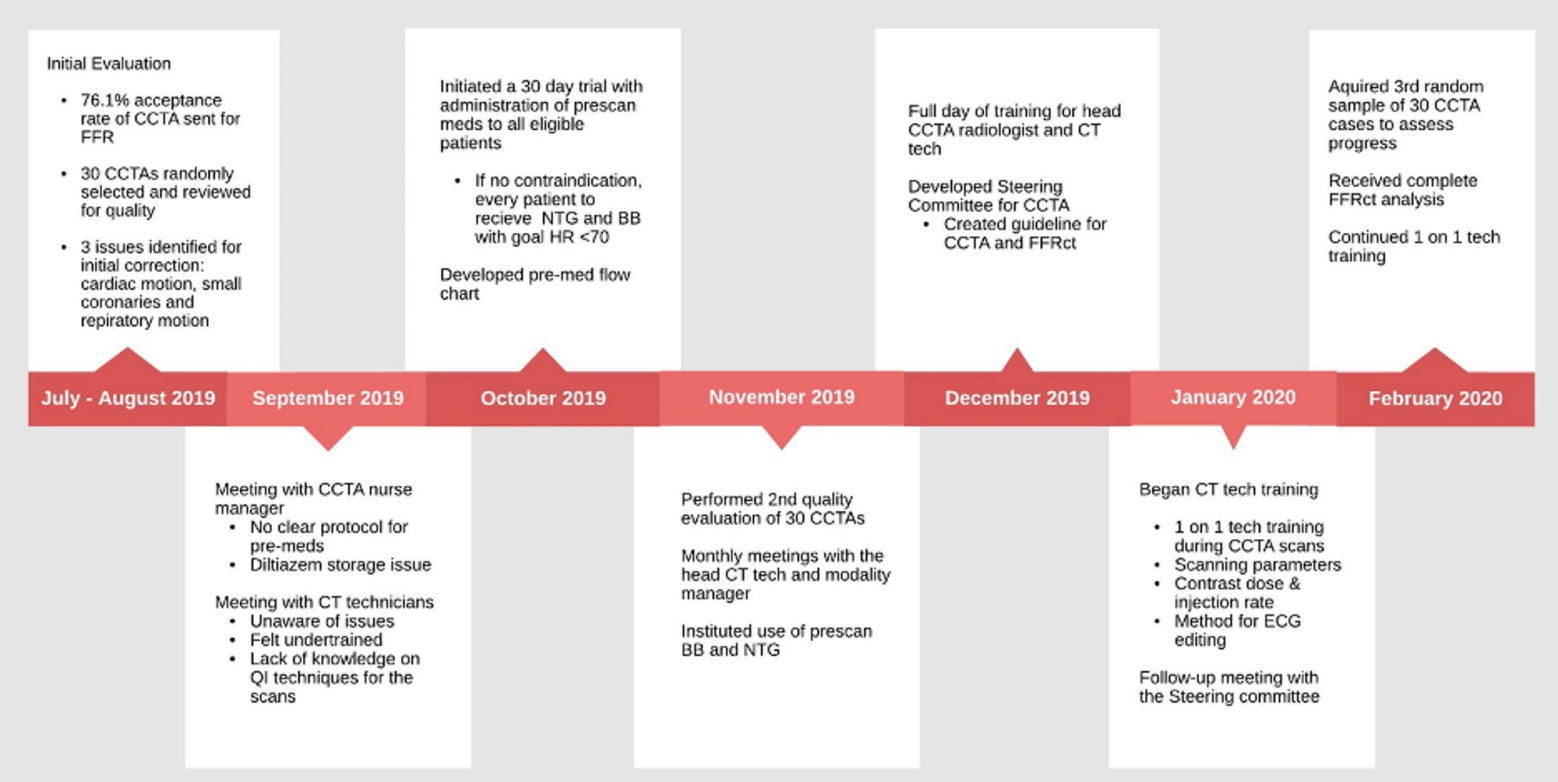
Illustration of the timeline employed for systematic intervention and periodic assessments. Further evaluation of the CCTAs was limited after February 2020 by the COVID-19 pandemic. BB, beta-blocker; CCTA, coronary CT angiography; FFRCT, fractional flow reserve derived from CT angiography; HR, heart rate; NTG, nitroglycerine

**Figure 3:**
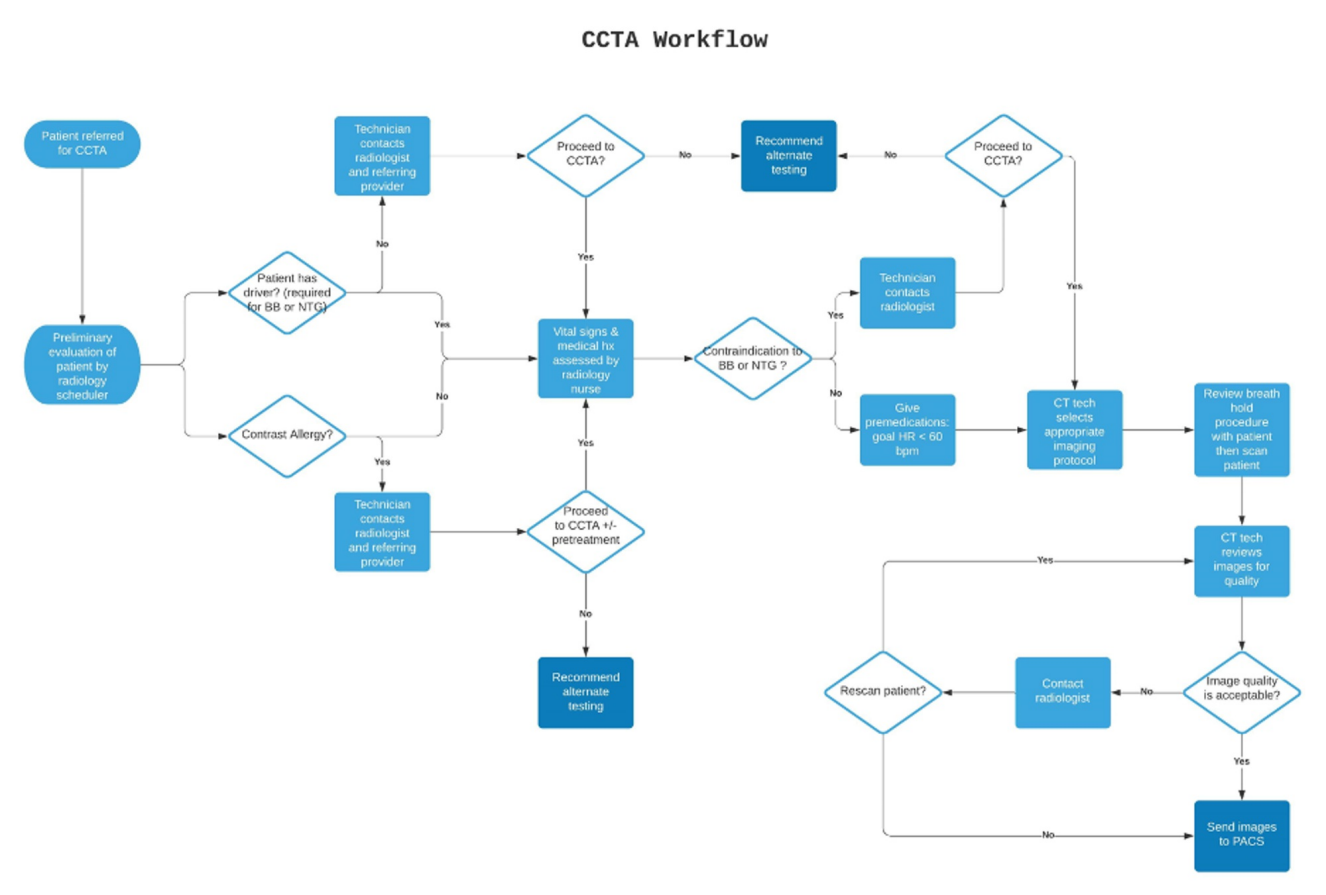
CCTA workflow. BB, beta-blocker; CCTA, coronary CT angiography; HR, heart rate; PACS, picture archiving and communication system; NTG, nitroglycerine

**Figure 4:**
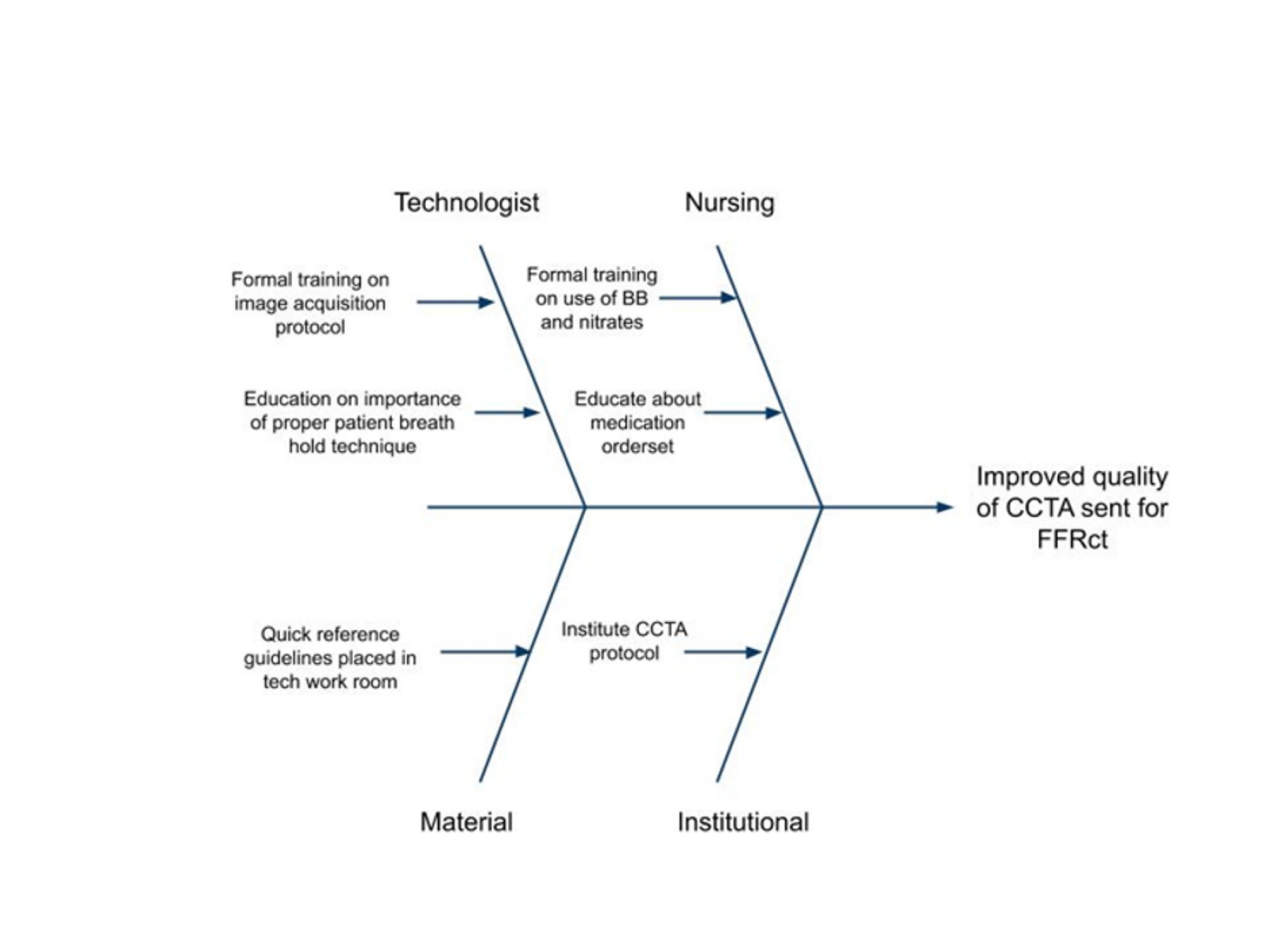
Fishbone diagram illustrating the individual steps that were improved upon for the CT technologist, nursing staff, input materials, and the institutional factors, all ultimately playing a part in the improved acquisition of CCTA studies for successful FFRCT analysis. BB, beta-blocker; CCTA, coronary CT angiography; FFRCT, fractional flow reserve derived from CT angiography

Figure 2 is an overall timeline demonstrating our intervention strategy and assessments, and Figure 3 demonstrates the overall CCTA acquisition workflow created. Figure 4 illustrates the individual improvement steps that were performed in order to reach our goal of improved CCTA acquisition for successful FFRCT analysis.

## Results

Pre-intervention CCTA review

Initial observations recorded by the expert cardiovascular radiologist of the first set of 30 random CCTA studies included cardiac motion (with an average recorded heart rate, at time of imaging, of approximately 70 beats per minute), small coronary arteries from lack of nitroglycerine administration, respiratory motion with inadequate breath hold, patient motion, contrast bolus timing and dose, blooming artifact and vessel opacification, and atrial fibrillation. Of the 30 studies, 14 were considered diagnostic studies and 16 non-diagnostic studies. Of the non-diagnostic studies, 8/16 (50%) had respiratory motion and 5/15 (31%) had cardiac motion with small coronary arteries. Our institution had an average FFRCT acceptance rate of 76.1%, whereas other top-performing sites that utilize FFRCT analysis have acceptance rates of >95% per month.

After initial review of the studies and identification of contributory factors to poor quality, interviews with the CT technologists and radiology nurses revealed several recurring issues:

 1. Limited training in CCTA acquisition and quality assessment of studies

 2. Limited knowledge in quality improvement techniques including ECG editing of CCTAs for atrial fibrillation or premature ventricular contractions, changing injection rates for large patients, and changing pitch and scan times, in addition to changing kV based on results of calcium scan

 3. No orders placed for nitroglycerine or metoprolol and subsequent inconsistent administration

 4. Hesitancy to make changes to the protocol

Analysis after medication administration education

The second analysis was performed to evaluate the effect of the metoprolol and nitroglycerine trial. The quality assessment again consisted of 30 CCTAs, this time with 21 diagnostic studies and only 9 studies with one or more poor quality coronary artery segments. Of the nine non-diagnostic studies, three (33%) were due to cardiac motion, five (55%) were due to respiratory motion, and one (11%) was due to suboptimal contrast bolus timing.

Given the success of the initial pre-scan medication trial at improving imaging quality, a standardized protocol was created and implemented by the in-house steering committee. One-on-one training with the CT technologists, including training for improved breath holds, was implemented to further improve protocol compliance.

**Figure 5:**
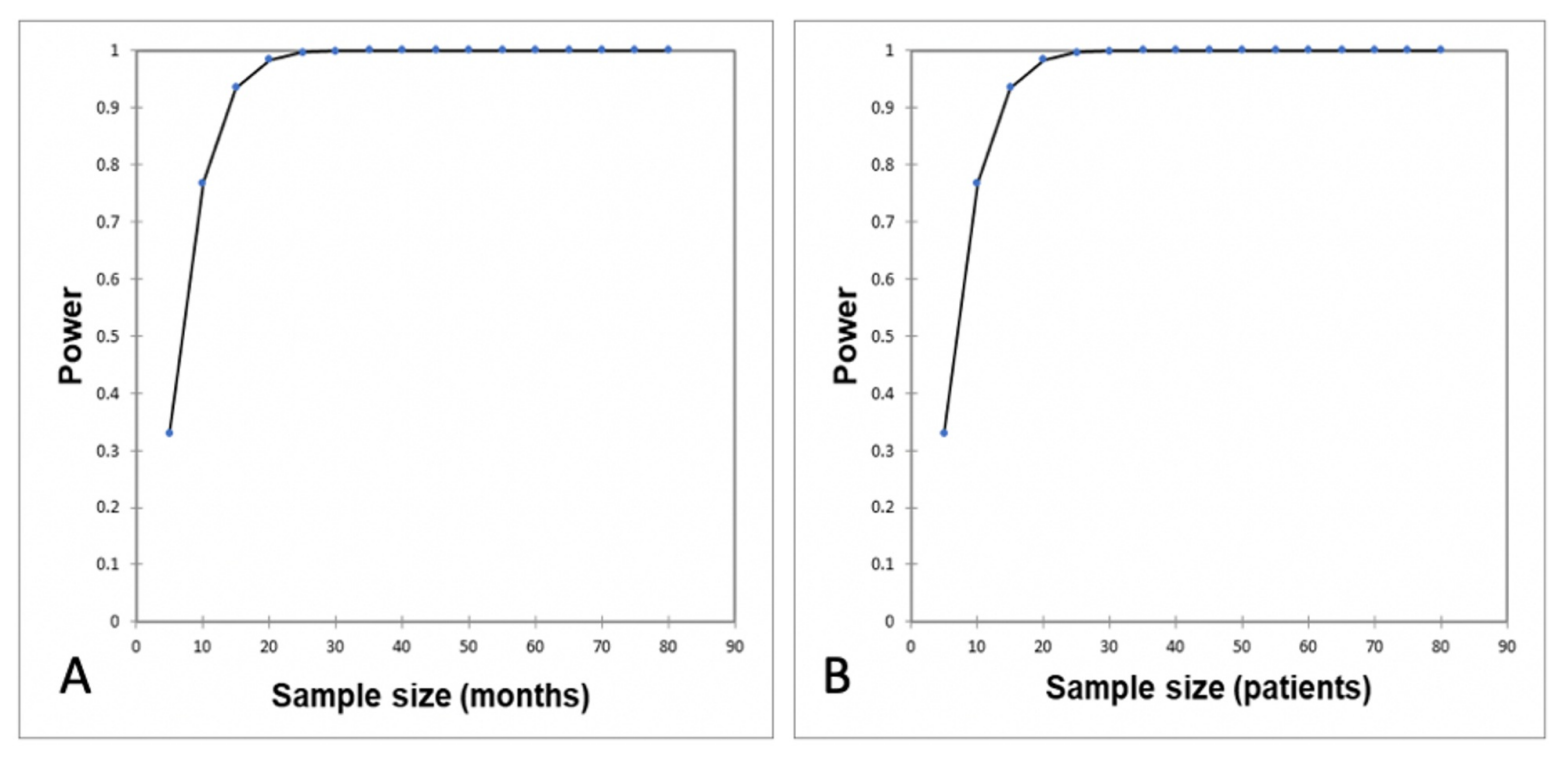
One-sample correlation power calculation for follow-up duration (A) and patient number (B). (A) One-sample correlation power calculation for follow-up duration with a power of 0.633 (R = 0.697), null hypothesis = no correlation. (B) One-sample correlation power calculation for patient number (n = 63) with a power of 0.99 (R = 0.697; R0 = 0).

**Figure 6:**
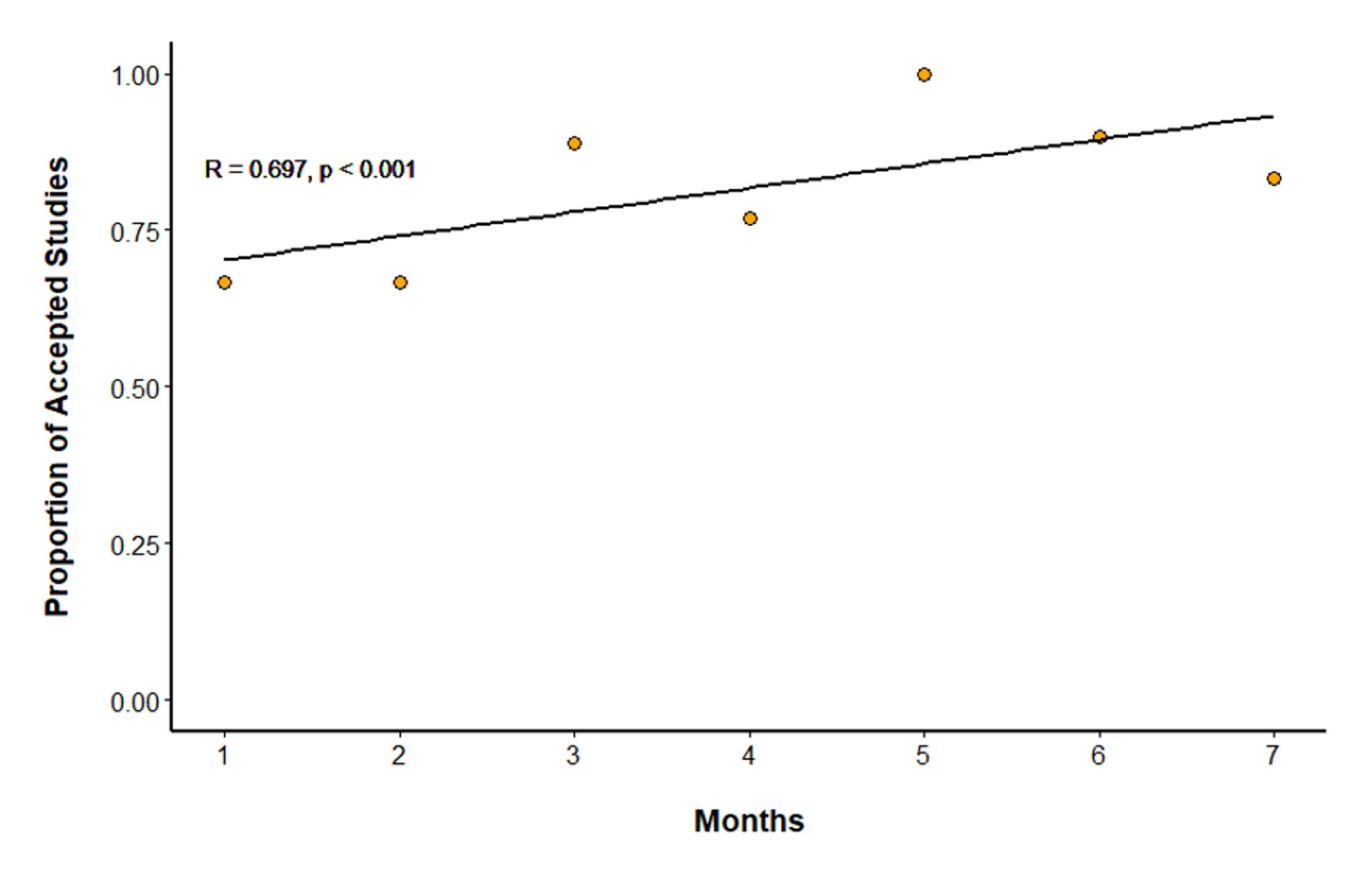
Linear weighted correlation using Spearman’s methods for the association of follow-up time with proportion of accepted studies. There is a moderately strong linear correlation between months after the initiative began and the proportion of accepted studies. R = 0.697 (95% CI: 0.522–0.815); R2 = 0.485. P < 0.001. Null hypothesis = no correlation.

One-sample correlation power calculation for follow-up duration revealed a power of 0.633, and the power calculation for patient number (n = 63) revealed a power of 0.99 (Figures 5A, 5B). A linear weighted correlation showed a moderately strong linear correlation between months after the initiative began and the proportion of accepted studies (R = 0.697; p < 0.001) (Figure 6).

**Figure 7:**
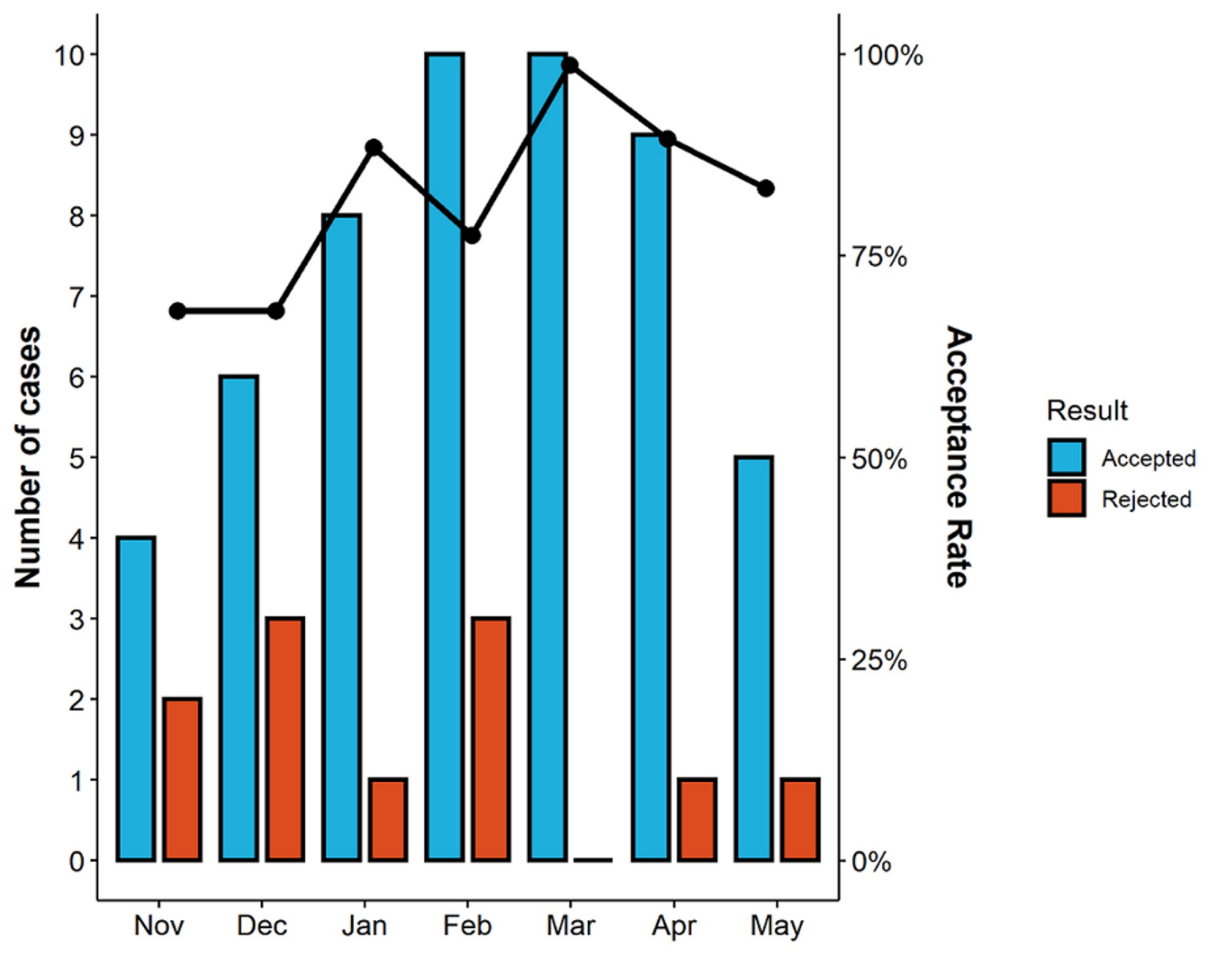
FFRCT analysis pre-intervention (November) through intervention phase and follow-up. Number of cases is represented by the bars (right-sided graph legend), and the acceptance rate is represented by the line graph (left-sided graph legend). Bar graph generated using  ggpubr: 'ggplot2' Based Publication Ready Plots, R package version 0.3.0 (https://CRAN.R-project.org/package=ggpubr).

Further FFRCT analysis was requested from pre-intervention until two months following the intervention (Figure 7). Our FFRCT acceptance rate increased to an average of 90% over the last three months of data collection.

## Discussion

The purpose of this study was to develop a step-by-step intervention designed to create a standardized CCTA acquisition for consistent and reliable FFRCT analysis. We proposed an intervention strategy created by consensus with key decision-makers in administration, radiology, cardiology, and the emergency department. The steering committee agreed upon creating a standardized pre-CCTA heart rate and vasodilator protocol, random CCTA quality spot checks, a clearly defined nursing checklist, and one-on-one training to CT technologists to improve the quality of the studies. By employing proper education and communication between multiple hospital departments, radiology nurses, and CT technologists, our institution rapidly improved coronary image quality to allow for high-level patient care.

FFRCT analysis

**Figure 8:**
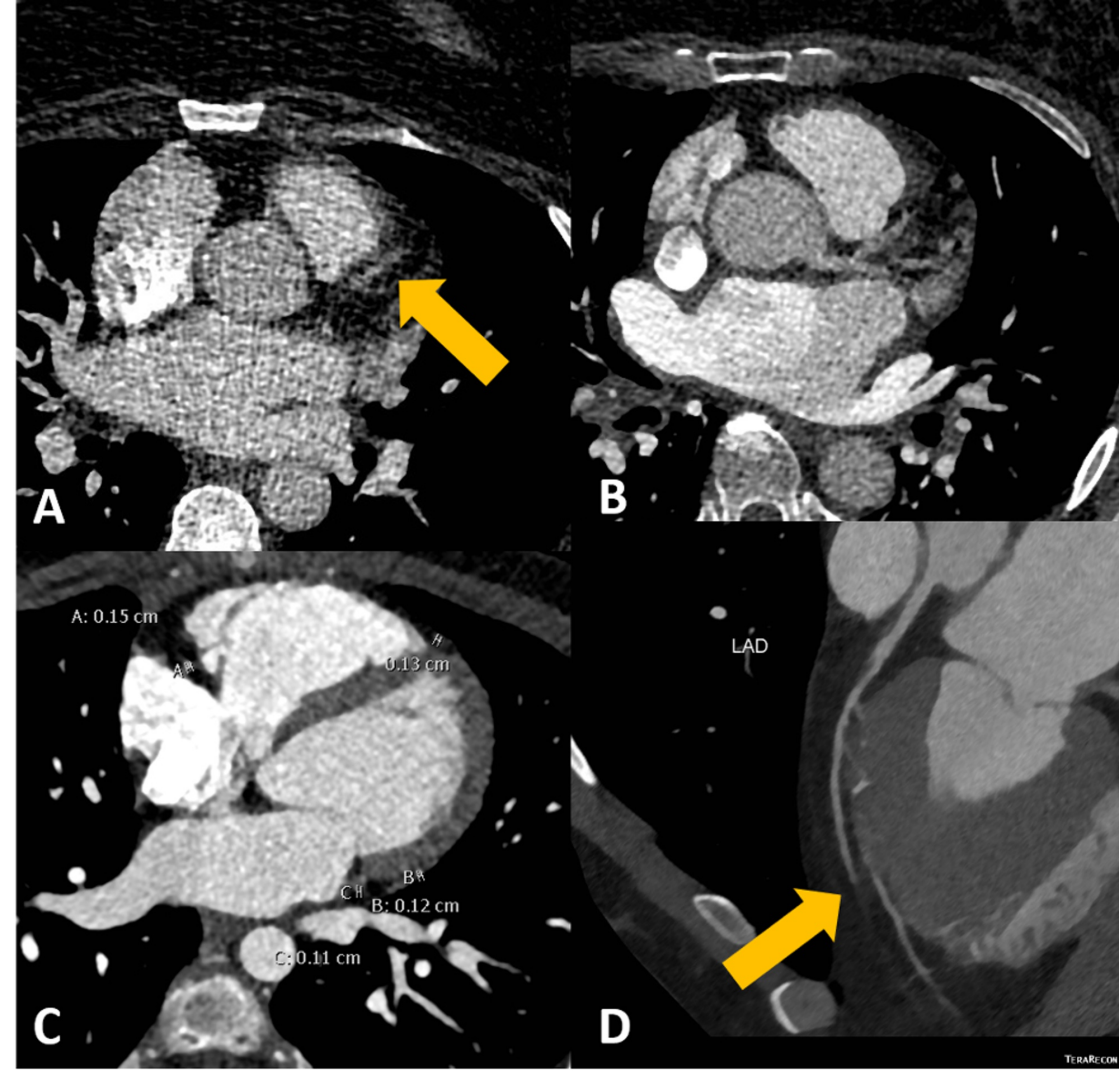
CCTA images demonstrating cardiac motion, respiratory motion, and poor opacification of coronary arteries. CCTA images demonstrating (A) extensive cardiac motion secondary to a high heart rate of greater than 70 beats per minute, poor contrast bolus timing, and quantum mottling artifact, resulting in poor visualization of the LAD (yellow arrow). (B) Poor contrast timing with the bolus of contrast predominately visualized in the left atrium. (C) Small coronary arteries without the administration of nitroglycerine. (D) Virtual reformat imaging demonstrating respiratory motion and an apparent break in the LAD (yellow arrow). All of these imaging factors limit the appropriate assessment of the coronary arteries. These examinations were subsequently rejected for FFRCT analysis. CCTA, coronary CT angiography; FFRCT, fractional flow reserve derived from CT angiography; LAD, left anterior descending artery

**Figure 9:**
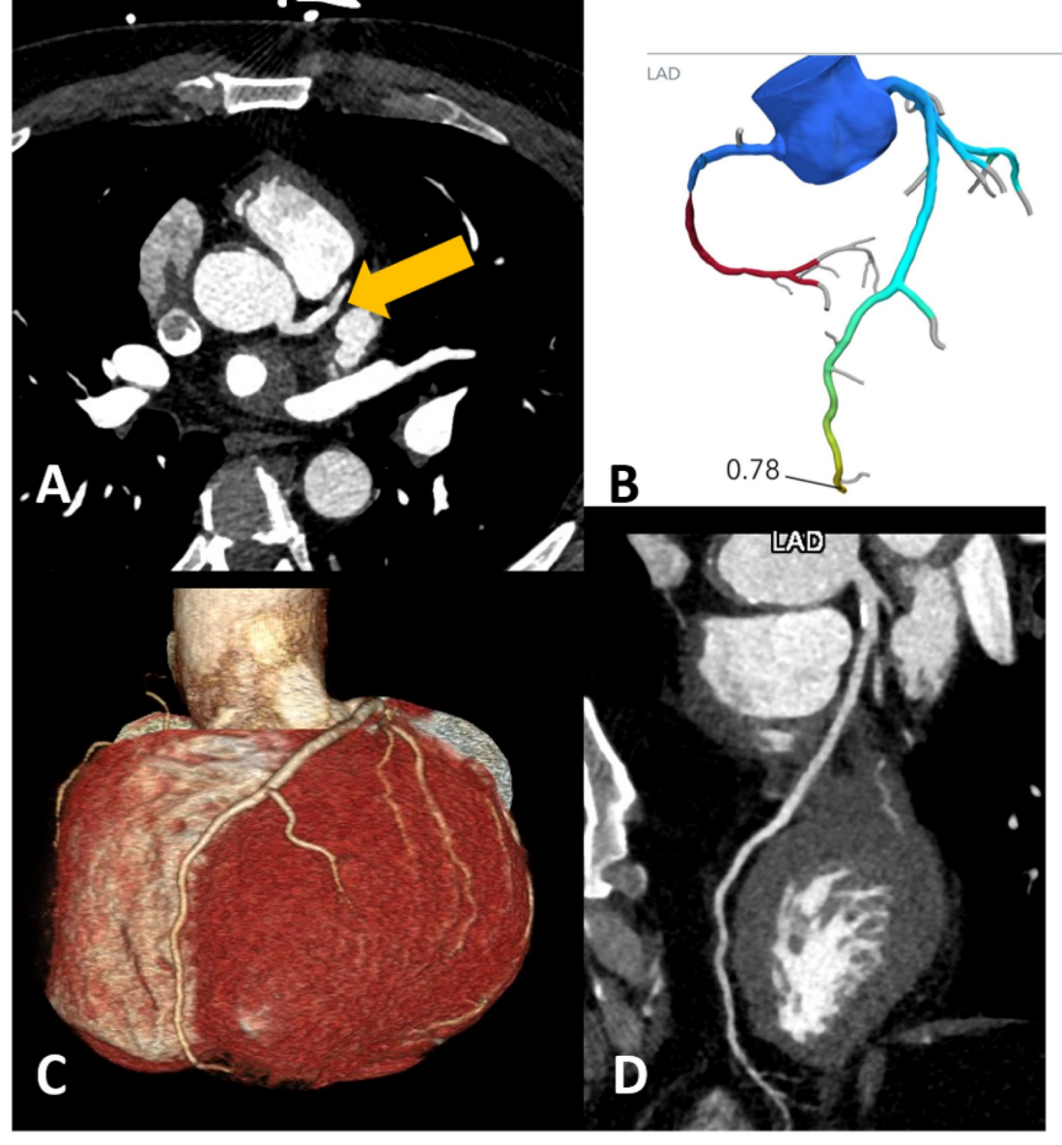
CCTA without cardiac motion, respiratory motion, and with properly dilated coronary arteries. (A) CCTA with properly opacified and dilated the left main coronary artery and LAD (yellow arrow). (B) Successful FFRCT analysis performed with values demonstrated for the LAD. (C) Three-dimensional reconstruction of the heart with a well-visualized LAD. (D) Virtual reformation of the LAD with minimal proximal stenosis from a calcified plaque. CCTA, coronary CT angiography; FFRCT, fractional flow reserve derived from CT angiography; LAD, left anterior descending artery

Multiple studies have demonstrated the overall utility of FFRCT in addition to CCTA in terms of risk stratification, added value, and prognostic significance [4,8-11]. However, the addition of FFRCT also adds an additional level of complexity to the overall study. Multiple factors must be well controlled for during the acquisition of the study to allow for detailed further software analysis including cardiac motion, coronary artery diameter, and respiratory motion (Figures 8A-8D, 9A-9D).

Initial rejection rates for FFRCT analysis reported throughout the literature range from 13% to 33%, with significant cardiac motion commonly cited as a reason for rejection [4,6]. Pontone et al. found that temporal resolution, section thickness, and heart rate are independent predictors of rejection for CCTA FFRCT analysis [5,9]. They postulated that their own study had a low rejection rate of 2.9% because of the use of dual-source technology and wide-coverage single-source scanners. While prior studies have shown that the use of nitrates and beta-blockers improves accuracy of the FFRCT analysis, overall analysis of success rates remain moving targets [12-14]. Vasodilation plays a role in improving overall analysis and visualization of the entire length of the coronary artery; however, this also must be balanced with decreased cardiac motion for quality assessment [14]. These studies emphasize that many factors must be optimized and controlled in order to produce a high-yield study that can be referred for FFRCT analysis, but specific protocols for imaging acquisition and specific factors influencing the analysis remain incompletely understood.

Limitations

We took a holistic approach to correcting the CCTA quality issues at our institution. Thus, we are limited in our evaluation regarding the extent to which each of the individual factors that we adjusted for affected the outcome measure of rejection/acceptance of FFRCT analysis.

A significant limitation present in this study is the sample size of each group. However, we felt that a random sample of 30 studies, representing approximately 30% of the CCTA studies/month, was an adequate representation. Continuing data acquisition of prospective patients sent for FFRCT analysis, as well as increasing the retrospective sample data, could increase the overall power of the study.

Finally, we do not want to underestimate the need for CT technologist and radiology nurse buy-in and re-training. When the issues were brought to their attention and they were subsequently given training, this likely controlled for many confounders that were problematic in FFRCT analysis acceptance rate and CCTA quality.

## Conclusions

Prior to intervention, our institution-specific overall FFRCT analysis acceptance rate was lower than average. Initial review demonstrated that 16 CCTA studies were deemed poor quality, of which eight were secondary to motion. Given that motion was a key contributory factor in visual quality inspection in addition to the success of FFRCT analysis, other unidentified factors needed to be controlled for in a holistic and protocol-driven manner.

Additional measures that were also taken during the education process, which certainly contributed to improved outcomes, were not directly assessed, including education on appropriate use of vasodilators. This likely resulted in better visualization of coronary vasculature. Moreover, a checklist was provided to the nursing staff to be completed prior to CCTA, which encouraged documentation of baseline cardiovascular status as well as relevant medical questions, factors that may affect or preclude the CCTA. Encouraging the nurses and technicians to call for any questions also worked to allow more open communication with the cardiac imagers, especially with more complex cases. 

Providing a standardized protocol for adequate heart rate control allowed us to decrease cardiac motion but also provided more strict acquisition parameters to control for other unknown variables, which proved to be an important aspect of improving the FFRCT analysis acceptance rate. In short, our holistic approach and newly issued standard protocol likely controlled for other factors, thus improving our FFRCT analysis acceptance rate.

## References

[1] Knuuti J, Wijns W, Saraste A (2020). 2019 ESC Guidelines for the diagnosis and management of chronic coronary syndromes. Eur Heart J.

[2] Wolk MJ, Bailey SR, Doherty JU (2014). ACCF/AHA/ASE/ASNC/HFSA/HRS/SCAI/SCCT/SCMR/STS 2013 multimodality appropriate use criteria for the detection and risk assessment of stable ischemic heart disease: a report of the American College of Cardiology Foundation Appropriate Use Criteria Task Force, American Heart Association, American Society of Echocardiography, American Society of Nuclear Cardiology, Heart Failure Society of America, Heart Rhythm Society, Society for Cardiovascular Angiography and Interventions, Society of Cardiovascular Computed Tomography, Society for Cardiovascular Magnetic Resonance, and Society of Thoracic Surgeons. Am Coll Cardiol.

[3] Tonino PA, Fearon WF, De Bruyne B (2010). Angiographic versus functional severity of coronary artery stenoses in the FAME study fractional flow reserve versus angiography in multivessel evaluation. J Am Coll Cardiol.

[4] Norgaard BL, Hjort J, Gaur S (2017). Clinical use of coronary CTA-derived FFR for decision-making in stable CAD. JACC Cardiovasc Imaging.

[5] Pontone G, Weir-McCall JR, Baggiano A (2019). Determinants of rejection rate for coronary CT angiography fractional flow reserve analysis. Radiology.

[6] Lu MT, Ferencik M, Roberts RS (2017). Noninvasive FFR derived from coronary CT angiography: management and outcomes in the PROMISE trial. JACC Cardiovasc Imaging.

[7] Cury RC, Abbara S, Achenbach S (2016). CAD-RADS(TM) Coronary Artery Disease - Reporting and Data System. An expert consensus document of the Society of Cardiovascular Computed Tomography (SCCT), the American College of Radiology (ACR) and the North American Society for Cardiovascular Imaging (NASCI). Endorsed by the American College of Cardiology. J Cardiovasc Comput Tomogr.

[8] Abdulla J, Asferg C, Kofoed KF (2011). Prognostic value of absence or presence of coronary artery disease determined by 64-slice computed tomography coronary angiography a systematic review and meta-analysis. Int J Cardiovasc Imaging.

[9] Chinnaiyan KM, Safian RD, Gallagher ML (2020). Clinical use of CT-derived fractional flow reserve in the emergency department. JACC Cardiovasc Imaging.

[10] Hlatky MA, De Bruyne B, Pontone G (2015). Quality-of-life and economic outcomes of assessing fractional flow reserve with computed tomography angiography: PLATFORM. J Am Coll Cardiol.

[11] Douglas PS, De Bruyne B, Pontone G (2016). 1-year outcomes of FFRCT-guided care in patients with suspected coronary disease: the PLATFORM study. J Am Coll Cardiol.

[12] Norgaard BL, Gaur S, Leipsic J (2015). Influence of coronary calcification on the diagnostic performance of CT angiography derived FFR in coronary artery disease: a substudy of the NXT trial. JACC Cardiovasc Imaging.

[13] Leipsic J, Yang TH, Thompson A (2014). CT angiography (CTA) and diagnostic performance of noninvasive fractional flow reserve: results from the Determination of Fractional Flow Reserve by Anatomic CTA (DeFACTO) study. AJR Am J Roentgenol.

[14] Ghekiere O, Salgado R, Buls N (2017). Image quality in coronary CT angiography: challenges and technical solutions. Br J Radiol.

